# PRL2 serves as a negative regulator in cell adaptation to oxidative stress

**DOI:** 10.1186/s13578-019-0358-z

**Published:** 2019-11-29

**Authors:** Xinyue Du, Yang Zhang, Xiao Li, Qi Li, Chenyun Wu, Guangjie Chen, XiaoKui Guo, Yongqiang Weng, Zhaojun Wang

**Affiliations:** 1 Department of Immunology and Microbiology, Shanghai Jiao Tong University School of Medicine, Rm 709 Bldg 5, 280 S. Chongqing Rd, Shanghai, 200025 People’s Republic of China; 2 Department of General Surgery, Huadong Hospital, Shanghai Medical College, Fudan University, Shanghai, 200040 People’s Republic of China; 3 Institute for Global Health, Shanghai Jiao Tong University School of Medicine-Chinese Center for Tropical Diseases Research, Shanghai, 200025 People’s Republic of China

**Keywords:** PRL2, Oxidative stress, Cell death, Inflammation, Ionizing radiation

## Abstract

High levels of ROS cause oxidative stress, which plays a critical role in cell death. As a ROS effector protein, PRL2 senses ROS and controls phagocyte bactericidal activity during infection. Here we report PRL2 regulates oxidative stress induced cell death. PRL2 senses oxidative stress via highly reactive cysteine residues at 46 and 101. The oxidation of PRL2 causes protein degradation and supports pro-survival PDK1/AKT signal which in turn to protect cells against oxidative stress. As a result, PRL2 levels have a high correlation with oxidative stress induced cell death. In vivo experiments showed PRL2 deficient cells survive better in inflammatory oxidative environment and resist to ionizing radiation. Our finding suggests PRL2 serves as a negative regulator in cell adaptation to oxidative stress. Therefore, PRL2 could be targeted to modulate cell viability in inflammation or irradiation associated therapy.

## Background

Reactive oxygen species (ROS) are generated during aerobic respiration in the mitochondria and in cellular response to xenobiotic, cytokines, and bacterial invasion [1, 2]. ROS include the superoxide anion (O_2_^−^), hydrogen peroxide (H_2_O_2_) and hydroxyl radicals (HO^·^) [1]. In physiological condition, ROS function as signals to promote cell proliferation and survival [3]. Severe increase of ROS, causing an imbalance between oxidants and antioxidants in favor of the oxidants, is referred as oxidative stress which results in damage of lipids, proteins and DNA. The accumulation of oxidative damage causes cell death and contributes to disease development [1].

The protein tyrosine phosphatase (PTP) family is a well-known ROS-effector protein family [4]. In the catalytic pocket, PTP proteins commonly possess a highly reactive cysteine (Cys) residue, which is susceptible to oxidation by ROS [4]. Phosphatases of regenerating liver (PRLs) belong to a subfamily of PTPs. They are dual specificity phosphatases, and have three members (PRL1, PRL2, and PRL3). PRLs are highly homologous, sharing at least 75% identity at the amino acid sequence level [5]. Among PRLs, PRL2 is the most abundantly and ubiquitously expressed in adult human tissues, suggesting its various roles in physiological processes [6]. Our previous work showed PRL2 is highly expressed in immune system [7]. In innate phagocytes, PRL2 rapidly responds to the oxidative stress at the inflamed site by down regulating its own expression, leading to enhanced respiratory burst. This positive feedback mechanism promotes bactericidal activity of phagocytes [7, 8]. Here we report PRL2 levels are highly related to the sensitivity of cells to hydrogen peroxide in vitro. PRL2 senses oxidative stress via highly reactive cysteine residues, which in turn to diminish its protein and support cell survival via PDK1/AKT pathway. In vivo, PRL2 deficient cells survive better under inflammatory environment and resist to ionizing radiation. Our finding may reveal a basic signal in cell adaptation to oxidative stress and PRL2 could be targeted to modulate cell viability in inflammation or irradiation associated therapy.

## Materials and methods

### Mice

The generation of PRL2 myeloid conditional knockout (*Ptp4a2*^*fl/fl*^*LysM*^*Cre*+^ B6) mice, PRL2 total knockout (*Ptp4a2*^*fl/fl*^*E2a*^*Cre*+^ B6) mice and their wild-type littermates (*Ptp4a2*^*fl/fl*^ B6) have been described previously [7, 8]. Wild-type C57BL/6 mice were purchased from Shanghai Laboratory Animal Center, Chinese Academy of Sciences. Wild-type C57BL/6 mice expressing CD45.1 were kindly provided from Junke Zheng’s Lab in Shanghai Jiao Tong University School of Medicine. Mice were housed in the Shanghai Jiao Tong University School of Medicine Animal Care Facilities under specific pathogen-free conditions.

### Primary cell isolation

Bone marrow cells were harvested from mouse femurs and tibias using phosphate buffer saline (PBS). After RBC lysis, cells were cultured in Dulbecco’s modified Eagle’s medium (DMEM) containing 10% heat-inactivated fetal bovine serum (FBS), 2 mM l-glutamine, and 100 U/ml penicillin/streptomycin (D10) resting for 1 h before further experiment. Primary mouse embryonic fibroblasts (MEFs) were isolated from E14.5 embryos according to the standard protocols [9].

### Plasmid construction

PRL2–pRK5 plasmid was generated as described previously [7]. The mutant PRL2 C46S, PRL2 C101S and PRL2 C164S were generated by PCR-based site-directed mutagenesis. PRL2-MigR1 was generated by cloning amplified PRL2 fragment into MSCV-based retroviral vector MigR1. All constructs were confirmed by DNA sequencing.

### Cell culture, transfection and infection

MEFs and HEK293T cells (ATCC) were cultured in D10. Transiently transfections were performed using Attractene transfection reagent according to manufacturer’s instruction (QIAGEN, Germany).

PRL2 overexpression stable cell lines were generated by retrovirus infection. To produce retroviruses, packaging cells (293T) were transfected with MSCV-based retroviral vector MigR1 containing gene of interest along with pVSVG and pCGP sequences using Attractene. Culture supernatants were collected and filtered 24 to 36 h post transfection. Target cells were infected with the filtered viral supernatants in the presence of 5 μg/ml polybrene (Sigma-Aldrich, USA) for 12 h, after which the medium was changed. Cells infected with MigR1 empty virus were used as control cell line. Following infection, cells were checked by fluorescence detection and western blotting.

### Cell treatment with hydrogen peroxide (H_2_O_2_)

Bone marrow cells used for viability assay under oxidative stress were seeded in 96-well plates at a concentration of 1 × 10^5^ cells in 100 μl medium. Used for immunoblotting were seeded in 12-well plates at a concentration of 3 × 10^6^ cells in 1 ml medium. Then bone marrow cells were treated with H_2_O_2_ (Sigma-Aldrich, USA) immediately after resting for 1 h. MEFs and 293T cells were seeded in 96-well plates at a concentration of 1 × 10^4^ cells in 100 μl medium or in 12-well plates at a concentration of 2 × 10^5^ cells in 1 ml medium. 24 h later, the cells in the wells were treated with indicated concentrations of H_2_O_2_ for indicated times. In some experiments, AKT phosphorylation was activated by 10 μM SC79 (CSN pharm, USA) for 0.5 h prior to H_2_O_2_ treatment.

### Cell viability assay

The cell viability was quantified by Cell Counting Kit-8 (YEASEN, China) according to the manufacturer’s instruction. In brief, the cells seeded in 96-well plates were incubated with the reagent 10 μl per well for 1 h under standard cell culture conditions and quantified by measuring absorbance at 450 nm. Data were normalized to control (100%) without stimulus, unless noted otherwise. Data of the growth curve were just shown in the form of absorbance at 450 nm.

### Expression and purification of recombinant PRL2

A pair of primers: 5′-AATTGAATTCTATGAACCGTCCAGCCCCT-3′ (forward) and 5′-GATCGGATCCCTACTGAACACAGCAG-3′ (reverse) were designed to amplify the target PRL2 gene. PRL2 gene was subcloned into the pET302/NT-His plasmid (Invitrogen, USA). And then the plasmid was transformed into *E. coli* BL21 cells for protein expression. Protein expression was initiated by IPTG and bacteria were harvested after 4 h culture. And then they were lysed and sonicated. The recombinant fusion protein His-PRL2 from *E. coli* lysates was purified by Ni–NTA Superflow Cartridges according to the manufacturer’s instruction (QIAGEN, Germany). The molecular weight and purity of recombinant proteins were identified by SDS-PAGE.

### In vitro H_2_O_2_ oxidation and DTT reduction

Recombinant protein His-PRL2 that dialyzed with PBS and adjusted to a final concentration of 4 mg/ml was incubated with various concentrations of H_2_O_2_ in a total volume of 20 μl for 20 min at room temperature. The reactions were stopped by adding 12.5 U of catalase (Sigma-Aldrich, USA) to consume H_2_O_2_. His-PRL2 reactions were further incubated with various concentrations of Dithiothreitol (DTT) for 20 min at room temperature. Following treatment, protein samples were denatured in non-reducing sample buffer (0.25 M Tris, pH 6.8, 10% SDS, 0.5% bromophenol blue, 50% glycerol). Protein oxidative and reductive states were separated on 12% SDS-PAGE by Coomassie Blue R-250 staining.

### Protein extraction and immunoblotting

Cells were harvested after treatment. After being washed by PBS twice, cells were lysed in 100 μl RIPA lysis buffer (Beyotime, China) supplemented with 0.1 mM PMSF on ice for 15 min. The oxidation state of PRL2 was protected by *N*-ethylmaleimide (Sigma-Aldrich, USA, 40 mM in lysis buffer). Protein concentration was determined by BCA assay (Sangon Biotech, China). Equal quantities of proteins were separated by SDS-PAGE under reducing or non-reducing conditions, transferred to a nitrocellulose membrane, and blotted with specific antibodies. The membrane was developed using Thermo SuperSignal reagent (Thermo, USA) and detected by ImageQuant LAS 4000 mini (GE, USA). Antibodies were used as followed: mouse anti-PRL2 antibody (Millipore, USA), mouse anti-GAPDH antibody (Sigma, USA), rabbit anti-phospho-PDK1 antibody (CST, USA), rabbit anti-phospho-AKT (Thr308) antibody (CST, USA), rabbit anti-phospho-AKT (Ser473) antibody (CST, USA), HRP conjugated mouse anti-Myc-Tag (CST, USA), HRP linked anti mouse IgG (CST, USA) and HRP linked anti rabbit IgG (CST, USA).

### The acute peritonitis model for studying myeloid cell survival at inflamed site in vivo

The acute peritonitis model was generated as described [7]. In brief, C57BL/6 wild-type mice (recipient mice) were given intraperitoneal injection of 5% thioglycollate (1 ml/mouse) to induce acute peritonitis. 24 h later, the recipient mice were exposed to total-body X-ray irradiation at a dose of 6 Gy. And then, carboxyfluorescein succinimidyl ester (CFSE)-labeled bone marrow cells (2 × 10^7^ per mouse) from PRL2^−/−^ CD45.2^+^ and wild-type CD45.1^+^ mice, mixed at 1:1 ratio were injected into peritoneal cavity of recipient mice. Recipient mice were sacrificed 24 h later and their peritoneal cells were collected. The peritoneal cells were stained with anti-CD45.1-PE (eBioscience, USA), anti-CD45.2-PerCP/Cy5.5 (eBioscience, USA) and anti-CD11b-APC (eBioscience, USA), and the frequencies of PRL2^−/−^CD45.2^+^CFSE^+^CD11b^+^ and PRL2^+/+^CD45.1^+^CFSE^+^CD11b^+^ cells were determined by flow cytometry.

### X-ray irradiation and blood cell count analyses

Female 6–8 week old PRL2 myeloid cell conditional knockout mice and their wild-type littermates were exposed to total-body X-ray irradiation at a dose of 9 Gy, which were administered at a dose rate of 1.89 Gy/min generated from RS-2000 Biological Irradiator (RadSource, USA). Circulating blood samples were collected from the mice tail for cell counting and peripheral blood smear at the indicated hours after irradiation.

### Statistical analyses

Data were presented as the mean ± SEM and all the differences were analyzed by unpaired Student’s *t* test or Analysis of Variance using GraphPad Prism software.

## Results

### PRL2 negatively regulates oxidative stress induced cell death

**Fig. 1:**
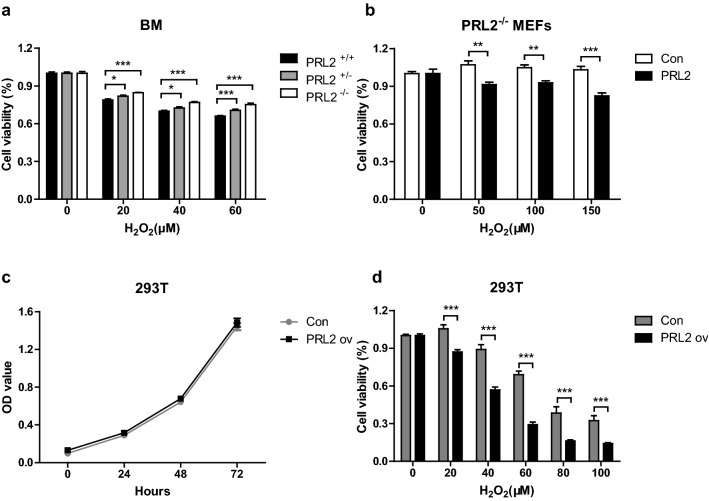
PRL2 negatively regulates oxidative stress induced cell death in vitro. **a** Relative cell viability of bone marrow cells from PRL2^+/+^, PRL2^+/−^ and PRL2^−/−^ mice was measured by the CCK-8 assay following oxidative stress induced by various concentrations of H_2_O_2_. **b** PRL2^−/−^ MEFs were transfected with pRK5. 24 h after transfection, relative cell viability was measured by the CCK-8 assay following oxidative stress induced by various concentrations of H_2_O_2_. **c** The growth curves of 293T stable cell lines with or without PRL2 overexpression were measured by the CCK-8 assay and shown in the form of absorbance at 450 nm. **d** 293T stable cell lines with or without PRL2 were treated with various concentrations of H_2_O_2_. Relative cell viability was measured by the CCK-8 assay. Error bars represent the SEM. Statistics were performed on pooled data from 3 independent experiments. *p < 0.05, **p < 0.01, ***p < 0.005

Oxidative stress induces cell death [10]. Our previous work showed PRL2 is sensitive to oxidative stress [7]. Therefore, we asked whether PRL2 is associated with oxidative stress induced cell death. To address this question, we used H_2_O_2_ to treat cells in vitro and undertook three approaches. First, we compared H_2_O_2_ induced cell death using myeloid cells from PRL2 wild-type, heterozygote and knockout mice. As shown in Fig. 1a, H_2_O_2_ treatment significantly induced bone marrow cell death. Under the same conditions, PRL2 WT cells died more than PRL2 heterozygote and deficient cells, while there is no difference at basal level (Additional file 1: Figure S1). Second, as a complementary approach, we transiently expressed PRL2 in mouse embryonic fibroblasts (MEFs) without PRL2. Consistent with previous results, we found that PRL2 reconstitution was associated with increased cell death following H_2_O_2_ treatment (Fig. 1b). Finally, we established PRL2 overexpression stable cell line using retroviral expression system. Overexpression of PRL2 alone does not affect cell proliferation (Fig. 1c), however, increase of PRL2 leaded to reduced cell viability under the oxidative stress (Fig. 1d).

### PRL2 senses oxidative stress via highly reactive cysteine residues

**Fig. 2:**
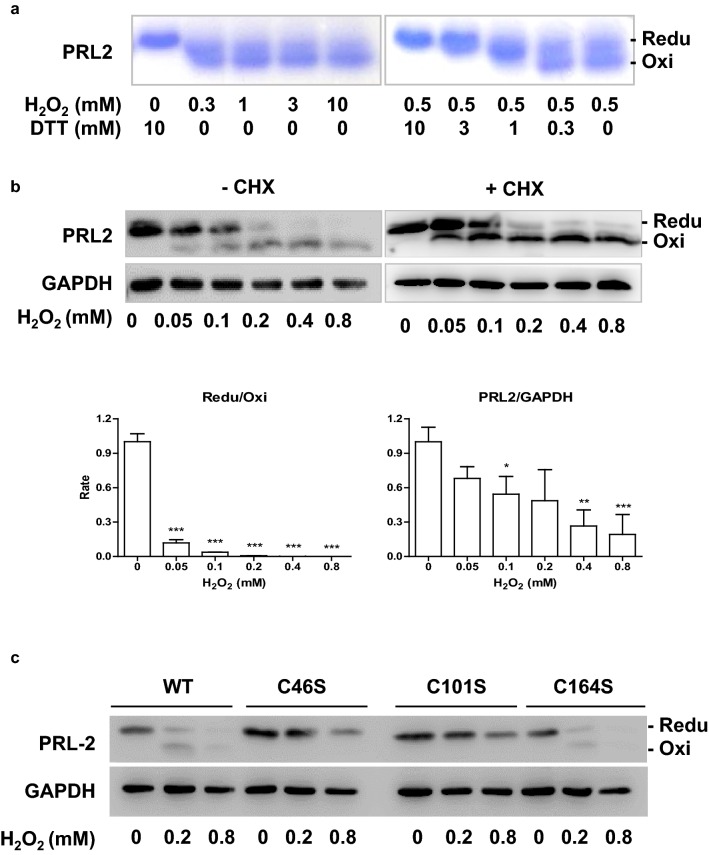
PRL2 senses oxidative stress via highly reactive cysteine residues. **a** PRL2 proteins were treated with DTT or H_2_O_2_ then were subjected to SDS-PAGE under reductive and oxidative conditions. **b** 293T cells were treated with various concentrations of H_2_O_2_ for 30 min. Some were pre-treated with cycloheximide for 1 h prior to oxidative stress exposure. Cell lysates were subjected to SDS-PAGE under reductive and oxidative conditions followed by immunoblot analysis with indicated antibodies. **c** 293T cells were transfected with pRK5. 24 h after transfection, cells were treated with various concentrations of H_2_O_2_ for 30 min. Cell lysates were subjected to SDS-PAGE under reducing and oxidative conditions followed by immunoblot analysis with indicated antibodies. The experiments in this figure were repeated at least three times with similar results. *p < 0.05, **p < 0.01, ***p < 0.005

It has been reported PRL1 can be oxidized by ROS to form an intramolecular disulfide bond between the catalytic Cys104 and the other conserved Cys49 residue [5, 11]. We proposed, by analogy, that PRL2 forms similar intramolecular disulfide bonding between cysteines. To test this hypothesis, we purified recombinant PRL2 protein from *E.coli* and performed SDS-PAGE analysis of PRL2 protein under reducing and oxidative conditions. As shown in Fig. 2a, PRL2 protein under reducing condition (10 mM DTT) yielded a single protein band. Meanwhile, PRL2 preincubation with H_2_O_2_ resulted in appearance of a second band with an increased electrophoretic mobility.

To determine whether the oxidative modulation of PRL2 also occurs in living cells, we examined endogenous PRL2 in 293T cells. While cells are treated with H_2_O_2_, there is the mobility shift in PRL2 corresponding to the transition between its reduced and oxidized forms (Fig. 2b, left panel). The oxidized form of PRL2 is initially detectable at 0.05 mM and becomes predominant at H_2_O_2_ concentrations exceeding 0.2 mM. Moreover, the total protein levels of PRL2 were significantly decreased with increasing concentration of H_2_O_2_. To examine whether this effect is associated with synthesis of new protein, we treated cells with 10 μg/ml cycloheximide 1 h prior to treatment with H_2_O_2_ and observed similar results (Fig. 2b, right panel).

To further test roles of cysteine residues in PRL2, we generated PRL2 mutants (C46S, C101S and C164S) in which individual cysteine residue was replaced with serine. We tested these mutant PRLs for their abilities to be modified by oxidation. WT PRL2, C46S, C101S and C164S PRL2 mutants were overexpressed in 293T cells respectively, and then cells were treated with different concentrations of H_2_O_2_. Unlike WT and C164S PRL2, the C46S and C101S PRL2 only showed single band following exposure to H_2_O_2_ (Fig. 2c). More importantly, C46S and C101S PRL2 mutants decreased less, as compared with WT and C164S PRL2. Above results suggest that C46 and C101 could participate in intramolecular disulfide bonding and are involved in protein degradation by oxidative stress.

### PRL2 negatively regulates cell survival under oxidative stress via PDK1/AKT signaling

**Fig. 3:**
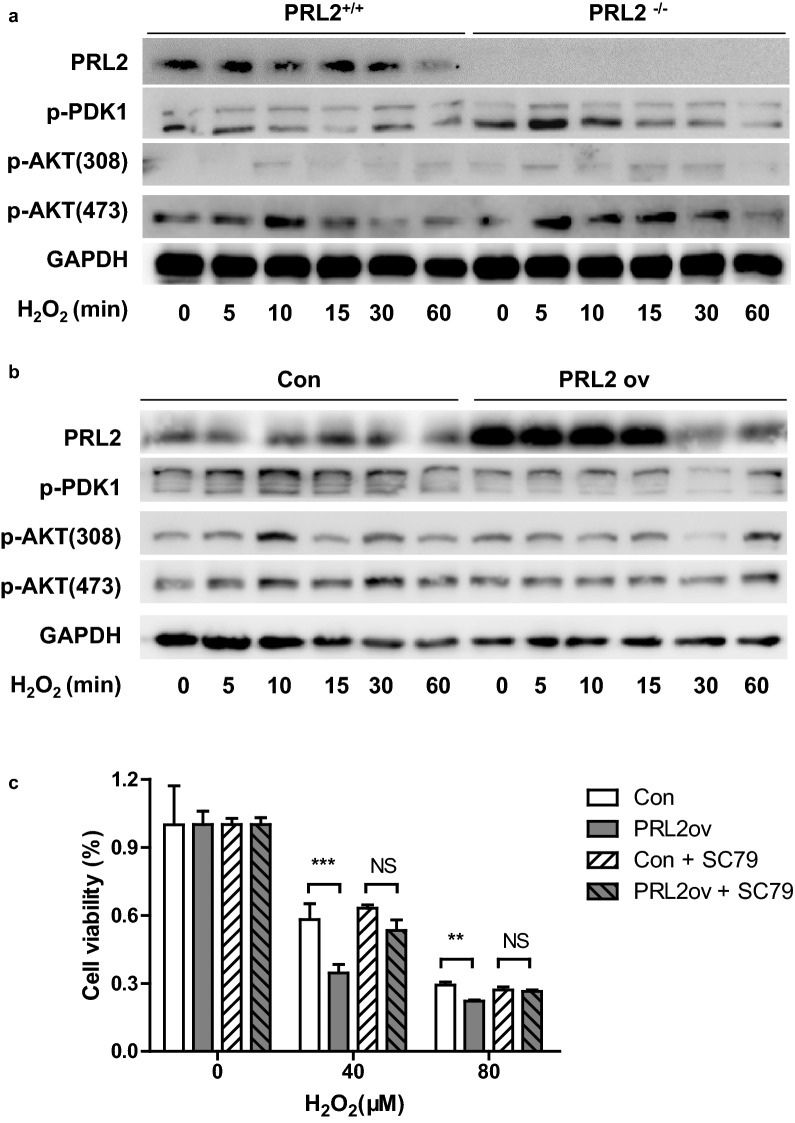
PRL2 negatively regulates cell survival under oxidative stress via PDK1/AKT signaling. **a** Bone marrow cells from PRL2^+/+^ and PRL2^−/−^ mice were treated with 50 μM H_2_O_2_ for different time. Cell lysates were subjected to SDS-PAGE followed by immunoblot analysis with indicated antibodies. **b** 293T stable cell line with or without PRL2 overexpression were treated with 50 μM H_2_O_2_ for different time. Cell lysates were subjected to SDS-PAGE followed by immunoblot analysis with indicated antibodies. **c** Prior to oxidative stress exposure, 293T cells were pre-treated with 10 μM SC79 (AKT activator) for 30 min. Relative cell viability was measured by the CCK-8 assay following oxidative stress induced by various concentrations of H_2_O_2_. The experiments in this figure were repeated at least three times with similar results. Error bars represent the SEM. Statistics were performed on pooled data from 2 independent experiments. **p < 0.01, ***p < 0.005

Oxidative stress is a combination of cellular damage and stress-responsive signaling [12, 13]. Compelling evidence suggests that oxidative stress-induced activation of the PI3K pathway is crucial for cell survival [14]. We examined the kinetic relationship of PI3K with PRL2 by testing the activation of PDK1 (3-phosphoinositide-dependent kinase 1) and AKT (also known as PKB), two major direct downstream effectors of PI3K. As shown in Fig. 3a, PDK1 activity was stimulated by H_2_O_2_ treatment in both PRL2 deficient and WT cells. Maximum levels of PDK1 activity were seen 5–10 min after addition of H_2_O_2_, followed by a return to basal levels at 60 min. H_2_O_2_ resulted in more significant activation of PDK1 in PRL2 deficient cells than that of WT cells. The activation of AKT on threonine 308 was consistent with the activation of PDK1. By contrast, when the 293T cells stably expressing PRL2 were exposed to H_2_O_2_, overexpression of PRL2 caused less activation of PDK1 and AKT (Fig. 3b).

To further determine whether PRL2 regulates oxidative stress-induced cell death through PDK1/AKT pathway, we manipulated the AKT activation using chemical activator, SC79. As shown in Fig. 3c, SC79 increased cell survival in PRL2 overexpression cells and abolished the difference between cell with and without PRL2 overexpression. Taken together, these results demonstrate that PRL2 might be positioned upstream of, and signal through, the PDK1/AKT pathway in the regulation of cell death.

### PRL2 deficient cells survive better in inflammatory environment

**Fig. 4:**
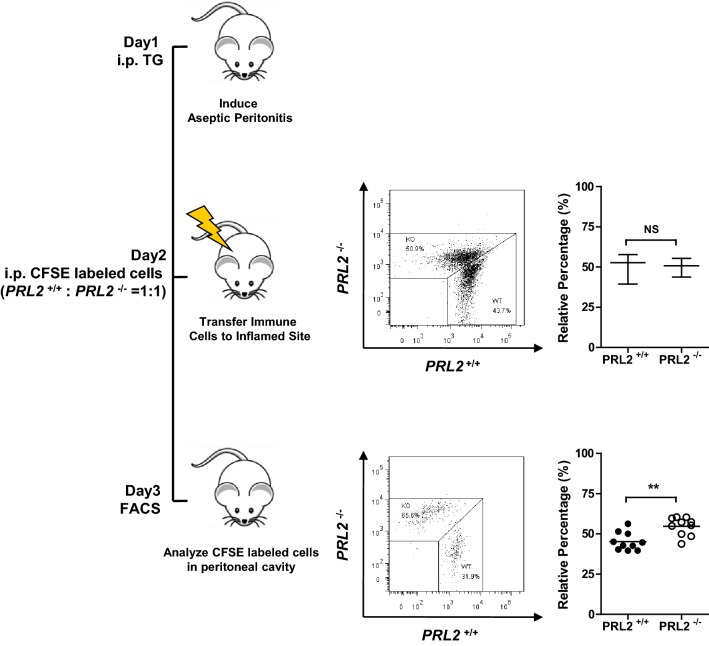
PRL2 deficient cells survive better in inflammatory environment in vivo. Peritonitis model of PRL2 on cell survival under inflammatory environment in vivo. The percentage of bone marrow cells was evaluated by flow cytometer assay. Error bars represent the SEM. Statistics were performed on pooled data from three independent experiments. **p < 0.01

In vitro experiments showed PRL2 levels are related to the sensitivity of cells to oxidative stress. To test the role of PRL2 in vivo, we compared the cell survival of PRL2 WT and deficient cells in inflammatory oxidative environment. In brief, aseptic inflammation was induced in the peritoneal cavity of recipient mice, and equivalent proportion of PRL2 WT and deficient cells were transferred into the same inflammatory cavity. One day later, significantly more PRL2-deficient cells than wild-type cells were left in the inflamed peritoneal cavity (Fig. 4). However, when mixed PRL2 WT and deficient cells were adoptively transferred to mice without peritonitis, no difference was observed (Additional file 1: Figure S2). Above results indicate that PRL2 deficient cells survive better in inflammatory environment.

### PRL2 deficient cells are resistant to ionizing radiation (IR)

**Fig. 5:**
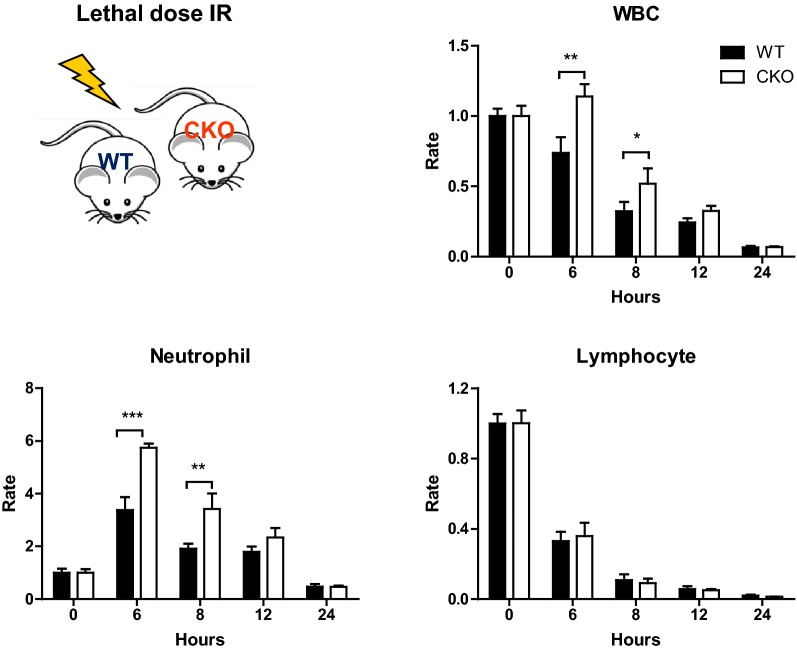
PRL2 deficient myeloid cells survive better after X-ray radiation exposure in vivo. PRL2 myeloid cell specific deficient mice (CKO) and their wide-type (WT) control mice were exposed to X-ray radiation at a dose of 9 Gy. Blood from tails was collected at the indicated hours after irradiation for cell counting and peripheral blood smear. Error bars represent the SEM. Statistics were performed on pooled data from 2 independent experiments. *p < 0.05, **p < 0.01, ***p < 0.005

IR is one of well-known exogenous stressors that promote oxidative stress and damage to cells [10]. To further test the role of PRL2 in living organism, we investigated the effects of IR on WT and PRL2 deficient cells in mouse model. PRL2 myeloid cell conditional knockout mice (CKO) and control mice (WT) were exposed to lethal dose IR. Peripheral blood samples were obtained and analyzed at 6, 8, 12 and 24 h after irradiation. The results showed the white blood cells (WBCs) count decreased after irradiation. At 6 and 8 h after irradiation, there were significant differences in the decrease of WBC count between WT and CKO mice. We further analyzed the blood cell types after IR exposure. There were significantly different in changes of neutrophils but not lymphocytes between WT and CKO mice (Fig. 5). Monocytes show the similar phenotypes as neutrophils (Additional file 1: Figure S3). Taken together, above results demonstrate that PRL2 deficient myeloid cells survive better after X-ray radiation exposure in vivo.

## Discussion

High levels of ROS lead to oxidative stress, which cause severe damage to the cell and form the basis of a wide variety of diseases [1]. Here we report PRL2 serves as a negative regulator in cell adaptation to oxidative stress. Our finding was supported by following evidences: (a) PRL2 deficient cells survive better in vivo and in vitro in oxidative environment; (b) PRL2 replenishment or overexpression promotes H_2_O_2_ induced cell death in vitro; (c) PRL2 negatively regulates ROS induced PI3K/PDK1/AKT signaling, a well-known pathway leading to cell survival.

Our previous work showed PRL2 is highly expressed in immune system. PRL2 senses ROS and controls phagocyte bactericidal activity by promoting respiratory burst [7]. ROS are used by the immune system as weapons against pathogens, however, ROS may also cause cell damage [15]. The finding in this study indicates that under inflammatory condition, decreased PRL2 in immune cells not only promotes ROS production but also protects cells against oxidative stress. Since PRL2 is expressed heavily in most of human tissues and cells [16], we speculate above mechanism exists wildly in human cells to survive in oxidative environment. It would be interesting to investigate the roles of PRL2 in ROS associated diseases, such as cancer, lung diseases or neural disorders.

Oxidative stress induced cell death is the result of the struggle between survival signaling and death signaling [1]. Our results indicate PRL2 senses oxidative stress via highly reactive cysteine residues. The oxidation of PRL2 causes protein degradation and supports pro-survival PDK1/AKT signal which in turn to protect cells against oxidative stress. PDK1 and AKT are two direct downstream effectors of PI3K [17]. Both of them possess a Pleckstrin Homology (PH) domain which interacts with phosphatidylinositol-3,4,5-triphosphate (PtdIns(3,4,5)P3), and phosphatidylinositol-4,5-bisphosphate (PtdIns(3,4)P2) [18, 19]. PDK1 and AKT co-localize at the plasma membrane, hence enabling PDK1 to activate AKT by phosphorylating Thr308 on its activation loop and promote cell survival [20]. In this study, we found AKT activator SC79 dramatically increased cell survival in PRL2 overexpression cells and abolished the difference between cells with and without PRL2 overexpression, suggesting PRL2 acts upstream of PIK3/PDK1/AKT. This observation is in line with our recent study that PRL2 negatively regulates Rac GTPase activation [7]. Rac and PI3K are intracellular signal transducers able to regulate multiple signaling pathways fundamental for cell behavior. They are tightly connected due to the ability to regulate each other in a lipid-dependent (PI3K upstream) or -independent (Rac upstream) manner [21]. It was reported that PI3K interacts with the GTP bound forms of Rac, via the p85 regulatory subunit, and serves as an effector of this GTPase [22].

Ionizing radiation induces direct generation of ROS in large quantities [23]. In present study, we found PRL2 deficient cells survive better in the irradiated animal. PRL2 myeloid cell conditional knockout and wild-type mice were exposed to a lethal dose of X-ray irradiation. Lymphopenia occurs following whole-body IR exposure. Changes in the counts of lymphocytes of CKO mice were similar with WT mice for any of the time points after irradiation. This result is in line with expectations, since the lymphocytes in CKO mice have similar PRL2 expression as WT mice. IR exposure also causes the decline in neutrophil counts. But the decline is preceded by an initial phase of granulocytosis, an event that is believed to be due to demargination of granulocytes and release of mature and early precursor for the relatively large pool of granulocytic cells within the bone marrow compartment [24]. 6–8 h after irradiation, more neutrophils appeared in the blood of CKO mice, suggesting without PRL2 neutrophils survive better under IR stress.

An increase of ROS can result in either cell survival or cell death [1, 25]. Preferential selection of survival signals lead to the protection of cells against damage induced by ROS, whereas preferential acceleration of death signals can be used to advantage in tumor therapy with oxidizing agents such as ionizing radiation and anticancer drugs [26]. Ever since PRL3 was found to be overexpressed in metastatic lesions derived from colorectal cancer [27], the PRLs family of phosphatases has received much attention. PRLs are considered as oncogenes that promote cell proliferation, migration, invasion, tumor growth and metastasis [5, 28, 29]. As a member of PRLs, PRL2 is reported that upregulated in many cancer (pancreatic, breast and lung cancer) and drive cancer metastasis [5]. However, there are also contradictory observations. PRL2 expression correlated with better survival in breast cancer patient [30] and high expression of PRL2 was a favorable prognostic marker in five independent breast cancer datasets [31]. Our finding indicates high expression of PRL2 is associated with ROS induced cell death which might contribute to cancer patient survival and response to ROS based chemotherapy or radiotherapy. Whether PRL2 promotes tumor progression or cell death depends on the kind of cells and tissues, the location of PRL2 and the sensitivity of PRL2 to oxidation. The further studies will be needed to reconcile the roles of PRL2 in cancer.

## Conclusion

Collectively, our study suggests PRL2 serves as a negative regulator in cell adaptation to oxidative stress via PDK1/AKT pathway. Thus PRL2 could be targeted to modulate cell viability in inflammation or irradiation associated therapy.

## Supplementary information

**Additional file 1: Figure S1.** The phenotypes of bone marrow cells from WT mice and PRL2 deficient mice. **Figure S2.** WT and PRL2 deficient cells survival in mouse peritoneal cavity without inflammation. **Figure S3.** PRL2 deficient monocytes survive better after X-ray radiation exposure in vivo.

## Data Availability

All relevant data are available in this published paper.

## Abbreviations

PRL2: phosphatases of regenerating liver 2
ROS: reactive oxygen species
Cys: cysteine
MEFs: mouse embryonic fibroblasts
DTT: dithiothreitol
IR: ionizing radiation
CKO: conditional knockout mice
WT: wild-type
WBCs: white blood cells
