# Supplementary material for: PRL2 serves as a negative regulator in cell adaptation to oxidative stress
Source: Cell Biosci. 2019 Nov 29;9:96. doi: 10.1186/s13578-019-0358-z (PMC6884919; doi:10.1186/s13578-019-0358-z)
Supplement: Supplementary file 1 — Additional file 1: Figure S1. The phenotypes of bone marrow cells from WT mice and PRL2 deficient mice. Figure S2. WT and PRL2 deficient cells survival in mouse peritoneal cavity without inflammation. Figure S3. PRL2 deficient monocytes survive better after X-ray radiation exposure in vivo. [file 13578_2019_358_MOESM1_ESM.doc]

**Additional Information**

**Figure S1. The phenotypes of bone marrow cells from WT mice and PRL2 deficient mice.**

**(a)** Bone marrow cells were isolated from PRL2^+/+^, PRL2^+/-^ and PRL2^-/-^ mice. Cell lysates were subjected to SDS-PAGE followed by immunoblot analysis with indicated antibodies. **(b)** Cell viability of bone marrow cells from PRL2^+/+^, PRL^2+/-^ and PRL2^-/-^ mice was measured by the CCK-8 assay and shown in the form of absorbance at 450 nm. **(c)** The population of myeloid cells from Wide type mice and PRL2 myeloid cell specific deficient mice were assessed by flow cytometry. Error bars represent the SEM. Statistics were performed on pooled data from 3 independent experiments.

**Figure S2. WT and PRL2 deficient cell survival in mouse peritoneal cavity without inflammation.**

CFSE labeled PRL2 WT and deficient cells were mixed (1:1) and adoptively transferred to mice without peritonitis. The percentage of bone marrow cells in peritoneal cavity was evaluated by flow cytometer assay. Error bars represent the SEM.

**Figure S3. PRL2 deficient monocytes survive better after X-ray radiation exposure *in vivo*.**

Wide type mice and PRL2 myeloid cell specific deficient mice were exposed to X-ray radiation at a dose of 9Gy. Blood from tails was collected at the indicated hours after irradiation for cell counting and peripheral blood smear. Error bars represent the SEM. Statistics were performed on pooled data from 2 independent experiments. *p<0.05.
